# Identification of serum miR-139-3p as a non-invasive biomarker for colorectal cancer

**DOI:** 10.18632/oncotarget.16171

**Published:** 2017-03-14

**Authors:** Lui Ng, Timothy Ming-Hun Wan, Johnny Hon-Wai Man, Ariel Ka-Man Chow, Deepak Iyer, Guanghua Chen, Thomas Chung-Cheung Yau, Oswens Siu-Hung Lo, Dominic Chi-Chung Foo, Jensen Tung-Chung Poon, Wai-Keung Leung, Roberta Wen-Chi Pang, Wai-Lun Law

**Affiliations:** ^1^ Department of Surgery, Li Ka Shing Faculty of Medicine, The University of Hong Kong, Hong Kong, China; ^2^ Centre for Cancer Research, Li Ka Shing Faculty of Medicine, The University of Hong Kong, Hong Kong, China; ^3^ Department of Medicine, Li Ka Shing Faculty of Medicine, The University of Hong Kong, Hong Kong, China

**Keywords:** miR-139-3p, miR-622, CRC, miRNA, biomarker

## Abstract

Aberrant levels of circulating microRNAs are potential biomarkers for the early detection of colorectal cancer. The aim of this study was to study miR-139-3p and miR-622 in serum as a non-invasive biomarker for colorectal cancer diagnosis. We applied quantitative polymerase chain reaction to determine the levels of miR-139-3p and miR-622 in 42 pairs of tumor and adjacent non-tumor tissues, and in serum samples of 117 patients and 90 control subjects. Our results showed that miR-139-3p was silenced whereas miR-622 was overexpressed in colorectal cancer. Similarly, serum miR-139-3p level was significantly lower in colorectal cancer patients than in control subjects whereas miR-622 was more frequently detectable in patients. ROC analysis showed that AUC of miR-139-3p was 0.9935, with a sensitivity of 96.6% and specificity of 97.8%. Serum miR-139-3p level showed high sensitivity and specificity for both early and late stage CRCs and proximal and distal CRCs. Detectable serum miR-622 showed a sensitivity of 87.5% and specificity of 63.5% for discriminating CRC patients, but the sensitivity dropped for late stage patients (72.7%). We also included analyses of the blood CEA level for comparing the diagnostic performance of these blood-based biomarkers. The median level in CRC patients (3.6 ng/ml) was significantly higher than that in control (1.8 ng/ml). The AUC value of CEA in diagnosing CRC patients was 0.7515. CEA showed a positive correlation with tumor stage and age of patients and its level was higher in male. Collectively, serum miR-139-3p has strong potential as a promising non-invasive biomarker in colorectal cancer detection.

## INTRODUCTION

Worldwide colorectal cancer (CRC) is the third most common cancer in men and the second in women. It is a leading cause of cancer related deaths and results in over 600 000 deaths annually [1–3]. Patient survival is highly dependent on the tumor stage at the time of diagnosis. The 5-year survival rate of CRC patients decreased from 90% for localized disease to 70% and 13% for regional and distant diseases, respectively [4], suggesting that the mortality rate can be greatly reduced if the patients can be diagnosed at earlier stage. However, due to the asymptomatic nature of CRC at early stages, less than 40% of CRC patients are diagnosed at localized stage [4]. Therefore, a reliable, economic and non-invasive approach is urgently in need to provide screening to patients at risk in order to improve the prognosis of patients with CRC.

MicroRNAs (miRNAs) are small non-coding RNA sequences of 19-25 nucleotides which function as post-transcriptional regulators of gene expression [5]. Many miRNAs which mediate cell growth and tumor progression have been found to be dysregulated in CRC [6]. Some of these dysregulated miRNAs are secreted into blood and can be detected in serum or plasma in highly stable form, hence circulating miRNAs have emerged as potential blood-based biomarkers for human cancers [7–9].

In this study, we investigated the levels of miR-139-3p and miR-622 in serum samples of CRC patients and control subjects, and evaluated their potential as non-invasive biomarkers for CRC screening.

## RESULTS

### Dysregulation of miR-139-3p and miR-622 in CRC tissues

**Figure 1:**
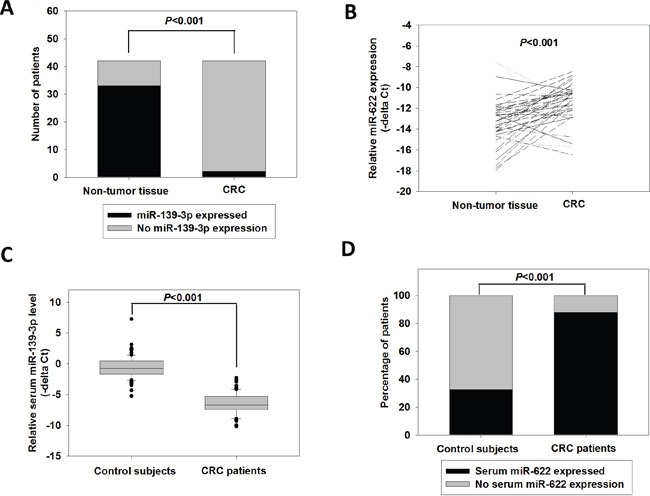
Expression of miR-139-3p and miR-622 in CRC tissue and serum samples **(A)** Number of CRCs and non-tumor tissues with detectable miR-139-3p level; p<0.001 (fisher exact test). **(B)** Relative miR-622 expression in 42 paired CRC and adjacent non-tumor tissues; p<0.001 (paired t-test). **(C)** Relative miR-139-3p expression in control subjects (N=90) and CRC patients (N=117); p<0.001 (Student's t-test). **(D)** Percentage of control subjects and CRC patients with detectable serum miR-622 level; p<0.001 (fisher exact test).

The expression levels of miR-139-3p and miR-622 were determined in 42 paired tumor and adjacent non-tumor tissue. We found that miR-139-3p was significantly silenced in CRC tissues (*P<*0.001). Among the 33 CRC patients who had miR-139-3p expression in their non-tumor tissues, only 2 of them showed detectable expression of this miRNA in their CRC samples (Figure 1A). Moreover, the miR-139-3p level of these two CRC samples was significantly lower than their paired non-tumor tissues (−14.835 vs −11.688, *P=*0.031). By contrast, miR-622 was significantly induced in CRCs when compared with paired non-tumor tissues (Figure 1B, −11.248 vs −13.460, *P*<0.001). miR-622 overexpression (at least 2-fold) was detected in 30 out of the 42 patients.

The correlation between miR-622 expression and patient's clinicopathological parameters was investigated. As indicated in Table 1, higher expression of miR-622 level was associated with advanced stage (−10.605 vs −11.614, *P*=0.036), lymph node metastasis (−10.739 vs −11.927, *P*=0.039) and distant metastasis (−10.461 vs −11.686, *P*=0.039), and its expression positively correlated with tumor stage (R=0.375, *P*=0.0143). These results revealed that miR-622 expression prominently correlated with CRC progression. We did not investigate the clinicopathological significance of miR-139-3p in CRC as its expression level was undetectable in most CRC tissues. Nonetheless, the significant dysregulation of miR-139-3p, and miR-622 as well, in CRC indicated their potentials as biomarkers for CRC.

**Table 1:** Clinicopathological correlation of miR-622 expression in CRC patients

		Relative miR-622 expression in CRC tissue−ΔCt (miR-622-RNU6)	p-value
Age	<55	−11.562	0.421
	>=55	−11.074	
Gender	Male	−10.987	0.298
	Female	−11.597	
Tumor Size	<=5	−11.286	0.877
	>5	−11.193	
Lymph node metastasis	Absent	−10.739	0.039*
	Present	−11.927	
Distant metastasis	Absent	−10.461	0.039*
	Present	−11.686	
Tumor stage	I to II	−10.605	0.036*
	III to IV	−11.614	

* indicates statistical significant difference between test parameters.

### Serum levels of miR-139-3p and miR-622 in CRC patients and control subjects

To investigate the potential of miR-139-3p and miR-622 as non-invasive biomarkers, the serum levels of miR-139-3p and miR-622 were detected in CRC patients and control subjects. As shown in Figure 1C, the serum miR-139-3p level in CRC patients was −6.500, which was significantly lower than that of the control subjects (−0.628, *P<*0.001). By contrast, we found that serum miR-622 was more frequently detected in CRC patients than in normal subjects (Figure 1D). It was detected in only 32.5% of normal subjects, but was detected in 87.8% of CRC patients (*P*<0.001).

### Evaluation of serum miR-139-3p and miR-622 as potential biomarkers of CRC

**Figure 2:**
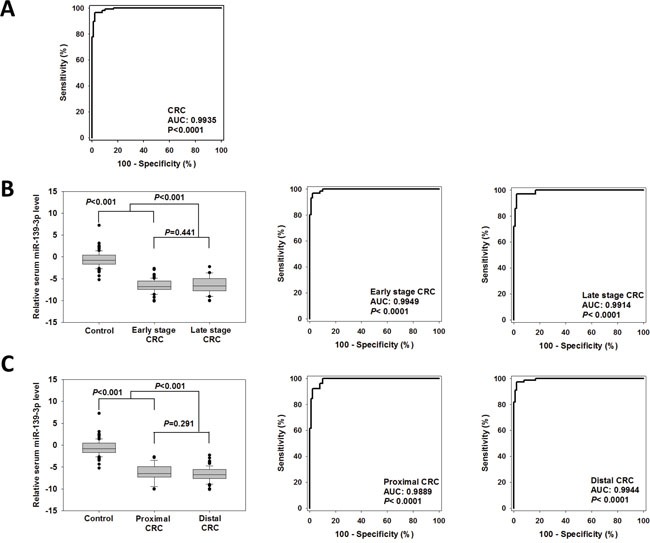
Diagnostic performance of serum miR-139-3p for CRCs **(A)** ROC curve was plotted to discriminate all CRC patients from control subjects. **(B)** Comparison of serum miR-139-3p level and diagnostic ability for early stage and late stage CRCs, and **(C)** proximal and distal CRCs.

Receiver operating characteristic (ROC) curve was plotted to evaluate the diagnostic accuracy of serum miR-139-3p. As shown in Figure 2A, miR-139-3p could well discriminate CRC patients from control subjects with area under the curve (AUC) value of 0.9935 (95%CI=0.9868-1.000, *P*<0.0001). The sensitivity and specificity at cutoff value of −3.524 were 96.6% and 97.8%, respectively, which met the 90/90 standard for cancer biomarkers [10]. The positive and negative predictive values were 98.3% and 93.6%, respectively. These statistics suggested that serum miR-139-3p had a high discriminating power to distinguish CRC patients from control subjects.

We also studied the diagnostic potential of serum miR-622. In the last section we showed that miR-622 was more frequently detected in serum samples of CRC patients when compared with normal subjects, hence we investigated whether detectable level of serum miR-622 indicated a higher risk of having CRC. The sensitivity and specificity of detectable serum miR-622 for diagnosing CRC were 87.8% and 67.5%, respectively. The positive and negative predictive values were 73.5% and 84.4%, respectively. These findings indicated that serum miR-622 was not accurate enough for CRC diagnosis.

### Diagnostic performance of serum miR-139-3p and miR-622 for CRC patients with tumors of different stages and locations

As shown in Figure 2B, there was no statistically significant difference in serum miR-139-3p level between early and late stage CRCs (−6.804 versus −6.633, *P=*0.441). ROC curves were plotted to evaluate the ability of serum miR-139-3p to screen early stage and late stage patients from controls. The AUCs in early and late stage CRC patients were 0.9949 and 0.9914, respectively; sensitivities were 96.7% and 97.2%, and specificities were both 97.8%, respectively. These findings suggested that serum miR-139-3p is a promising biomarker for both early and late stages of CRC. The performance of these potential diagnostic biomarkers was also compared between patients with CRCs at distal or proximal regions (Figure 2C). There was no statistically significant difference in serum miR-139-3p level between patients with proximal CRC than those with distal CRC (−6.622 vs −6.215, *P*=0.291). The AUCs of miR-139-3p in distal and proximal CRCs patients 0.9944 and 0.9889, respectively. Their sensitivities were 97.4% and 92.3%, respectively, and specificities were both 97.78%, indicating that serum miR-139-3p is a promising biomarker for both early and late stages of CRC.

There was also no significant difference in serum miR-622 level between early and late stage CRCs (Figure 2C, −6.693 versus −6.158, *P=*0.558); however, the sensitivities of serum miR-622 in diagnosing early and late stage CRCs were 90.0% and 72.7%, respectively, indicating that the diagnostic performance of serum miR-622 level dropped in late stage CRC patients. There was no significant difference in serum miR-622 level between patients with proximal or distal CRCs (−6.290 versus −6.730, *P=*0.582). The sensitivities of serum miR-622 for diagnosing CRCs at proximal and distal regions were similar (88.9% and 89.5%, respectively).

Furthermore, we examined whether these biomarkers were affected by demographic factors (age and gender). Serum miR-139-3p level showed no significant correlation with age and gender. Serum miR-622 level showed no correlation with gender, but its expression increased with age (R=0.511, *P*=0.003). The serum miR-622 level in patients above 65 years old was ∼2.5 fold of those below (−6.206 vs −7.543, *P*=0.045).

It has been suggested that blood carcino-embryonic antigen (CEA) level could serve as a biomarker for diagnosing CRC, hence we retrieved from our hospital's database the data of CEA level of the same serum samples for comparison on the diagnostic performance. The CEA level in CRC patients (median: 3.6 ng/ml) was significantly higher than that in control subjects (median: 1.8 ng/ml, p<0.001). Further evaluation on this potential biomarker showed that the AUC value of CEA in diagnosing CRC patients was 0.7515 (95%CI= 0.6864−0.8166, *P*<0.0001). The sensitivity and specificity at cutoff value of 2.5 ng/ml were 65.2% and 71.0%, respectively; and 33.9% and 96.8% at 5 ng/ml. The positive and negative predictive values at 2.5 ng/ml were 73.5% and 62.3%, respectively; and 90.9% and 54.3% at 5 ng/ml. The sensitivity decreased while the specificity increased for higher CEA cutoff value. Furthermore, the blood CEA level positively correlated with tumor stage (R=0.281, p=0.008). The sensitivities of CEA in diagnosing early stage and late stage CRC patients at 2.5 ng/ml were 54.7% and 77.8% and at 5 ng/ml were 26.4% and 50%, respectively, indicating that CEA showed a higher sensitivity in patients with higher stage CRC. This finding is in line with previous reports that the sensitivity of CEA for early colon cancer patients is low and increases with an increasing stage of the disease [11, 12]. Blood CEA level also correlated with age of overall population (R=0.309, p<0.0001), and was significantly higher in male when compared to female (median 2.5 vs 1.8 ng/ml, p=0.007). On the other hand, blood CEA level was not affected by tumor location.

These results indicated that serum miR-139-3p level was a promising and independent biomarker for CRC screening and showed high sensitivity in CRC patients of different tumor locations and stages.

## DISCUSSION

In this study, we showed that serum miR-139-3p was a promising biomarker for screening CRC patients with a high AUC (0.9935), sensitivity (96.6%) and specificity (97.8). It is quite rare for a single biomarker to achieve such good performance in distinguishing cancer patients from normal subjects. Our results revealed that there was a large difference in 139-3p level between CRC and normal, at both tissue and serum levels. MiR-139-3p was expressed in adjacent normal mucosa samples of 33 CRC patients, whereas loss expression of this miRNA was detected in 31 of them, and the remaining 2 patients also displayed ∼8-fold reduction of miR-139-3p level in their CRC specimens, indicating that miR-139-3p was significantly silenced or repressed during CRC development. In line with its repression in CRC in comparison to paired non-tumor tissue, serum miR-139-3p level in CRC patients was also significantly lower than that in control subjects, with an average of ∼64-fold reduction. These results consistently demonstrated that miR-139-3p expression was significantly repressed at both tissue and serum levels of CRC patients, providing an explanation to the high performance of this potential biomarker in identifying CRC patients. This study demonstrated that serum miR-139-3p showed higher AUC, sensitivity and specificity when compared to other potential biomarker such as blood CEA. In addition, serum miR-139-3p showed high AUC and sensitivities in both early and late stage CRCs and CRCs at proximal and distal locations, thus serum miR-139-3p is able to compensate the limitation of sigmoidoscopy and FOBT that show lower sensitivity for proximal CRC [13] and some blood-based biomarkers, for example, blood CEA that show lower sensitivity for early stage CRC [14]. In addition, serum miR-139-3p showed no correlation with age or gender, whereas in this study we showed that blood CEA level was affected by age and gender. These results suggested that serum miR-139-3p could be an additional screening test for patients in whom other conventional screening might show false-negative or false-positive results.

One limitation of this study is patients with colorectal polyps were not included for comparison. It is worth investigating whether serum miR-139-3p level can identify this type of patients from normal subjects. If it can, this biomarker could help to detect patients with high risk of polyps so that they can have their polyps surgically resected before it is progressed into cancer, which aids in reducing the prevalence of CRC. On the other hand, if serum miR-139-3p level in patients with colorectal polyps was similar to that of normal subjects, it can still be used to identify high-risk CRC patients who require further colonoscopy confirmation, so that surgical operation can be applied to those showing positive findings as early as possible. Hence, further study on the serum level of this miRNA in patients with colorectal polyps is warranted to determine its potential application.

Notably, certain types of patients might not be appropriate for this screening approach, for example, patients with adrenocortical carcinoma, bladder carcinoma *in situ* and adrenal pheochromocytomas in which dysregulation of miR-139-3p in tissue level has been reported [15–17]. Moreover, though miR-139-3p repression in CRC was demonstrated in this study and the others [18, 19], high miR-139-3p was on the other hand associated with liver metastasis in another study [20]. Therefore, further studies investigating the serum miR-139-3p level in patients with diseases mentioned above are warranted to improve the precision of this biomarker.

Previously, Kanaan et al reported a panel of plasma miRNAs including miR-139-3p and two other miRNAs (miR-431 and miR-15b distinguished Stage IV CRC from controls with an [AUC = 0.896 (95% CI: 0.78−1.0)] [21]. Their study demonstrated a 25-fold change in miR-139-3p level comparing CRC and control plasma samples, whereas our study demonstrated a 64-fold change in serum samples comparing CRC and control subjects. This greater discrepancy in serum miR-139-3p level provides an explanation to the higher AUC, sensitivity and specificity obtained in our study. We surmise two factors contributing to such difference. Firstly, Kanaan et al's study examined the level of miR-139-3p in plasma whereas we detected its level in serum. Secondly, the ethnic background of the sample population in their study and our study was different. Both these factors have been reported to affect the miRNA level. Wang et al reported that the serum samples always showed higher miRNA concentrations than plasma and caution must be taken when comparing miRNA data generated from different sample types [22]. Zhao ZH et al. had revealed the profiles of circulating miRNAs in plasma samples from Caucasian American women or that from African American with early breast cancer were inconsistent, and separate case-versus-control comparisons stratified by race were necessary [23]. Since this study did not compare miR-139-3p level between serum and plasma samples, nor among samples of different race, we could not conclude their effects on miR-139-3p level. However, we believed that the diagnostic performance in different sample types and ethnic background should also be determined for potential miRNA biomarkers in the future.

In line with Balaquer F et al's study which reported miR-622 as one of the most significantly upregulated miRNAs in CRC [24], this study showed that miR-622 was significantly overexpressed in CRCs and high expression associated with lymph node and distant metastases. On the other hand, another study showed that miR-622 was down-regulated in CRC and miR-622 level was lower in metastatic CRCs than in non-metastatic counterparts [25]. These contradictions suggest that the role of miR-622 in CRC is more complicated than expected. Notably, though we reported serum miR-622 was more frequently detectable in CRC patients than in control subjects, the mean level of this serum miRNA was on the other hand higher in normal subjects when comparing the control and CRC individuals with detectable level of serum miR-622. Since this study aimed at investigating the diagnostic potential of serum miRNAs for CRC, we did not further look into the underlying mechanism of miR-622 overexpression or repression in CRC. Nevertheless, we believed that a comprehensive investigation of the regulatory mechanism of miR-622 in CRC could improve our knowledge on miRNA biology as such contradictory observations have also been reported for other miRNAs [26–28].

In conclusion, we demonstrated that the miR-139-3p level in both tissue and serum samples was significantly lower in CRC patients, and its serum level showed a high sensitivity and specificity for CRC diagnosis. Further validation can be performed for this serum miRNA or in combination with other miRNAs which showed high potential for CRC screening to form a detection panel, in order to detect the disease of CRC patients as early as possible so that their prognosis will be improved.

## MATERIALS AND METHODS

### Sample size calculation

The sample size required for this diagnostic test was calculated using the characteristics represented by the sensitivity and specificity of serum miRNA to detect CRC. According to our pilot study which included 72 CRC patients and 48 control subjects, the sensitivity and specificity of miR-139-3p were both ∼95%. Hence, around 93 CRC patients and normal subjects will be required to obtain the lower limit of the 95% confidence interval of both the sensitivity and specificity to be > 85% [29].

### Patient tissue and blood samples

The study included 42 paired tumor and adjacent non-tumor tissues from CRC patients who had their tumor specimens resected at the Department of Surgery, Queen Mary Hospital, HKSAR, between the years 2009 and 2011. All samples were flash-frozen in liquid nitrogen, and stored at −80°C until further molecular analysis. This study also included pre-operative serum sample from 117 CRC patients between 2011 and 2016 and serum sample from 90 control subjects between 2013 and 2016 who showed no cancer and colorectal polyp in their colonoscopy examination. Serum was collected following centrifugation of blood at 400 x g for 10 min. The supernatant was transferred to new 1.5-ml centrifugation tubes and stored at −80°C. Written consent was received for all patients whom we recruited. The clinicopathological data was obtained from the hospital records relating to age, gender, diagnosis, tumor location and size, TNM staging, local invasion, differentiation and distant metastasis. This study was approved by the Institutional Review Board of the hospital.

### RNA extraction and quantitative RT-PCR

Total RNA was extracted from tumor tissue and adjacent normal mucosa or serum sample (250 μl) using mirVana® miRNA isolation Kit (Ambion, Austin, TX) according to the manufacturer's instructions. The RNA concentrations were measured using Nanodrop ND-1000 spectrophotometer (Nanodrop Technologies, Wilmington, DE, USA). This total RNA was used to reverse transcribe mRNA complementary DNA (cDNA) and miRNAs using TaqMan Microarray Assays (Applied Biosystems, Foster City, CA). Each reverse transcriptase reaction utilized 10 ng of total tissue RNA or 3.33 μl total serum RNA, 0.15 μl dNTP (100 mM total), 1.00 μl Multiscribe RT enzyme (50 U/μl), 1.50 μl 10X RT buffer, 0.19 μl RNase Inhibitor (20 U/μl) and 3 μl 5X RT primer making a total reaction volume to 15 μl with nuclease-free water. TaqMan microRNA assay (Applied Biosystems, Foster City, CA) were used to relatively quantify and detect the miRNA levels of miR-139-3p, miR-622; U6 and RNU48 were utilized as internal controls. The reversely transcribed cDNA was diluted 1:20 before the qPCR reaction volume was mixed and 1.33 μl was combined with 10 μl 2X Universal PCR Master Mix (no AmpErase UNG), 7.67 μl water and 1.0 μl 20X MicroRNA Assay. A total volume of 20 μl per reaction was transferred to a 96-well MicroAmp plates (Applied Biosystems, Foster City, CA) and incubated for 10 min at 95°C, followed by 40 cycles at 95°C for 15 sec. then 60°C for 60 sec. All samples were run in duplicates. Samples with cycle threshold (Ct) over 40 were regarded as no expression. The relative miRNA level was expressed as −ΔCt, which is calculated by the formula: −(Ct of miR-139-3p minus Ct of U6).

### Statistical analysis

The comparison of miRNA level between paired-tumor and non-tumor tissues was done by paired t-test or Fisher exact test. The serum miRNA levels of CRC patients and control subjects were compared using Student's t-test or Fisher exact test. Associations between miRNA expression and clinicopathological features were explored using Student's t-test, Mann–Whitney U test or Spearman correlation. All relationships of the relative expression of the target miRNA were performed using SigmaStat 3.5 (Systat Software Inc., San Jose, CA, USA) and *P<* 0.05 was considered to be statistically significant.

## Abbreviations

CRC: colorectal cancer
